# Efficacy and safety of oncolytic viruses in advanced or metastatic cancer: a network meta-analysis

**DOI:** 10.1186/s12985-021-01630-z

**Published:** 2021-07-31

**Authors:** Ruiyang Xie, Xingang Bi, Bingqing Shang, Aiping Zhou, Hongzhe Shi, Jianzhong Shou

**Affiliations:** 1 Department of Urology, National Cancer Center/National Clinical Research Center for Cancer/Cancer Hospital, Chinese Academy of Medical Sciences and Peking Union Medical College, Panjiayuan Nanli #17, Chaoyang District, Beijing, 100021 China; 2 Department of Medical Oncology, National Cancer Center/National Clinical Research Center for Cancer/Cancer Hospital, Chinese Academy of Medical Sciences and Peking Union Medical College, Chaoyang District, Beijing, China

**Keywords:** Oncolytic virus, Advanced or metastatic cancer, Network meta-analysis

## Abstract

**Background:**

Oncolytic viruses (OVs) have shown prospects in advanced and metastatic cancer, and many clinical trials have been carried out. To compare OV therapies comprehensively and provide a categorized profile and ranking of efficacy and safety, a network meta-analysis was conducted.

**Methods:**

A total of 5948 studies were screened and 13 randomized controlled trials with 1939 patients, of whom 1106 patients received OV therapies, comparing four OVs (NTX-010, pexastimogene devacirepvec (Pexa-Vec), talimogene laherparepvec (T-VEC), and pelareorep) were included in a Bayesian network meta-analysis. Eligible studies reported at least one of the following clinical outcome measures: objective response rate (ORR) and grade ≥ 3 adverse events.

**Results:**

Compared to systemic treatments alone, talimogene laherparepvec (T-VEC) (OR 7.00, 95% CI 1.90–26.00) and T-VEC plus systemic treatment (2.90, 0.80–11.00) showed better objective response rates (ORRs), whereas Pexa-Vec 1 * 10^9^ pfu plus systemic treatment (0.91, 0.26–3.00) and pelareorep plus systemic treatment (1.10, 0.61–2.00) were found to be comparable. The grade ≥ 3 adverse event ranking of the treatments from worst to best was as follows: T-VEC (ranking probability 24%), Pexa-Vec 1 * 10^9^ pfu plus systemic treatment (21%), Pexa-Vec 1 * 10^9^ pfu (17%), T-VEC plus systemic treatment (13%), pelareorep plus systemic treatment (13%), systemic treatments (18%), Pexa-Vec 1 * 10^8^ pfu (12%), and NTX-010 (20%).

**Conclusions:**

Compared with other oncolytic virus therapies for patients with advanced or metastatic cancer, T-VEC and T-VEC plus systemic treatment appear to provide the best ORR therapy in terms of monotherapy and combination respectively, but should be given with caution to grade ≥ 3 adverse events. Conversely, combining OVs with chemotherapy or target agents was demonstrated not to improve efficacy compared with chemotherapy or target agents alone. Combining OV therapies with immune-checkpoint inhibitors, instead of chemotherapy or target agents, tended to provide better ORRs without causing severe adverse events. This study will guide treatment choice and optimize future trial designs for investigations of advanced or metastatic cancer.

**Supplementary Information:**

The online version contains supplementary material available at 10.1186/s12985-021-01630-z.

## Introduction

Oncolytic virus (OV), a new therapeutic approach to cancer treatment, is capable of replicating preferentially within tumour cells and inducing immunogenic cell death [1]. Initially, direct tumour oncolysis (apoptosis, necrosis, and autophagy) was considered the dominant mechanism [1]. However, the induction of systemic antitumour immunity, promoted by the direct lysis and release of tumour-associated antigens, appeared to be a critical element that mediated the immune response. The release of local cytokines (for example, tumour necrosis factor-α, interferon-γ, and interleukin-12) and additional cellular danger-associated molecular patterns (DAMPs; for example, heat shock proteins, high mobility group box 1 protein, ATP, and uric acid) played a role in enhancing innate and adaptive immune responses against tumour cells, which also explained the regression of distant tumours that were not injected with or exposed to OVs in a previous study [2].

Current comprehensive treatments for cancers include surgery, radiotherapy, chemotherapy, targeted therapy, immunotherapy, and so on. However, conventional monotherapies have met the challenge of resistance and drug discontinuation due to toxicity. With various approaches, combination therapies have been demonstrated to improve efficacy and cancer management [3]. Generally, an eligible OV selected for potential therapy was either natural or artificially modified. To date, three OVs in total have been approved for patients with advanced cancers: Rigvir, an RNA virus for melanoma treatment [4]; H101, an adenovirus for the treatment of nasopharyngeal carcinoma [5]; and talimogene laherparepvec (T-VEC), a herpes simplex virus for the treatment of unresectable recurrent melanoma [6]. The species of OVs enrolled in ongoing or completed clinical trials include adenovirus, coxsackievirus, herpes simplex virus, Maraba virus, reovirus, measles virus, vesicular stomatitis virus, Newcastle disease virus, and Seneca Valley virus [7]. A variety of malignancies in different systems have been targeted in OV clinical trials, including melanoma, gastrointestinal cancers, lung cancers, head and neck cancers, genitourinary cancers, breast and gynaecological cancers, and sarcomas [7].

The combination therapy of OVs and other antitumour treatments is recognized as a new attempt in the era of immunotherapy. Although several meta-analyses have demonstrated the efficacy and safety of oncolytic viruses, a comprehensive network meta-analysis describing individual ranking and optimal combination of the available OVs is absent. Therefore, we conducted this network meta-analysis of OV therapy to provide clinicians with information on the optimal options for their patients.

## Methods

The Preferred Reporting Items for Systematic Reviews and Meta-Analyses (PRISMA) guidelines were followed in the network meta-analysis (Additional file 1: table S1) [8]. The Bayesian model for network meta-analysis was applied in this study. The Institutional Review Boards of the Chinese Academy of Medical Science and Peking Union Medical College approved the study.

### Database searching and study screening

Articles in all languages published up to February 20, 2021, including those in Embase, PubMed, the CENTRAL registry of the Cochrane Library, and ClinicalTrials.gov, were searched. The major search protocol consisted of the terms "oncolytic virus", "oncolytic therapy", and "cancer" (Additional file 1: table S2).

### Criteria for study selection

The inclusion criteria for study selection were as follows:

1. Phase II/III randomized controlled trials with eligible published or unpublished results
2. Trials that enrolled patients who were cytologically or histologically diagnosed with cancer
3. Trials with an intervention arm including an oncolytic virus
4. Trials reporting at least one of the following clinical outcomes or adverse events:
  The objective response rate (ORR), defined as the ratio of the sum of patients with a partial response to the sum of patients with a complete response to treatment
  All adverse events were referred to in the Common Terminology Criteria for Adverse Events (CTCAE) version 5.0 [9].

Trials that reported results from subgroup analysis with stratified patient groups and potential bias were excluded.

In cases in which studies contained early and updated forms of data, the most recent results were used and extracted from the study for which data updates were available, regardless of their status on the ClinicalTrials.gov website. Literature such as cohort studies, case reports, and letters were all excluded, whereas conference abstracts were included and screened. Initial screens focused on titles and abstracts, and the full text of articles was secondarily assessed for final inclusion.

### Data extraction and assessment of bias risk

General characteristics, including study ID, sample size, patient age, patient sex, intervention arm, control arm, and virus species were extracted. Data for each outcome were extracted from the intention-to-treat population. Reported adverse events of any grade were included, except those mentioned only in severe events to avoid potential selective reporting bias.

The risk of bias of each study was assessed with the Cochrane Risk of Bias Tool and divided into high, unclear, or low risk of bias. The following categories were scored: random sequence generation, allocation concealment, the blinding of participants and personnel, the blinding of outcome assessment, incomplete outcome data, selective reporting, and other biases (Additional file 1: figure S1).

### Data synthesis and statistical analysis

Odds ratios (ORs) were used to describe rate outcomes, including ORR and adverse event data, with 95% confidence intervals (95% CIs). Several treatments were stratified as systemic treatments to obtain an appropriate sample size, including chemotherapy (paclitaxel, carboplatin, pemetrexed, FOLFOX6, irinotecan, and docetaxel), immunotherapy (ipilimumab and granulocyte–macrophage colony-stimulating factor (GM-CSF)), and targeted agents (sorafenib and bevacizumab).

Network meta-analyses of ORRs and adverse events were conducted in the Bayesian random-effects consistency model, where all indirect comparisons were taken into account to arrive at a single, integrated, estimate of the effect of all included treatments based on all studies. We estimated the ranking probability of the different treatments for ORRs and adverse events using surface under the cumulative ranking curve (SUCRA) analysis [10]. Finally, heterogeneity among studies was assessed by comparing the mean difference and I^2^ values if more than one trial existed. The variance of the consistency and inconsistency model was estimated by comparison.

In R 4.0.2 with the “GeMtc” and “rjags” packages, network plots of ORR and adverse events were generated to illustrate the sample size and number of trials (https://www.r-project.org/) [11]. Furthermore, analyses of heterogeneity were conducted in R. To determine heterogeneity effects, the number of adaptations was set to 5000, whereas the sample iteration parameter was adjusted to 10,000. The network meta-analyses of ORRs and adverse events, as well as the ranking probability analysis, were conducted in ADDIS software (version 1.16.6) [12].

To assess the reliability of the study results, we planned two sensitivity analyses. The first analysis for ORR excluded the Bradbury et al. study (NCT01708993) [18] because four arms were stratified into two arms and detailed data were not applicable. The second analysis for grade ≥ 3 adverse events excluded the Noonan et al. study (NCT01280058) [14] due to the 100% rate of grade ≥ 3 adverse events in both arms. A relatively high risk of bias in the selective reporting of outcomes was observed in these two studies.

## Results

### Systematic review and characteristics of enrolled trials

**Fig. 1:**
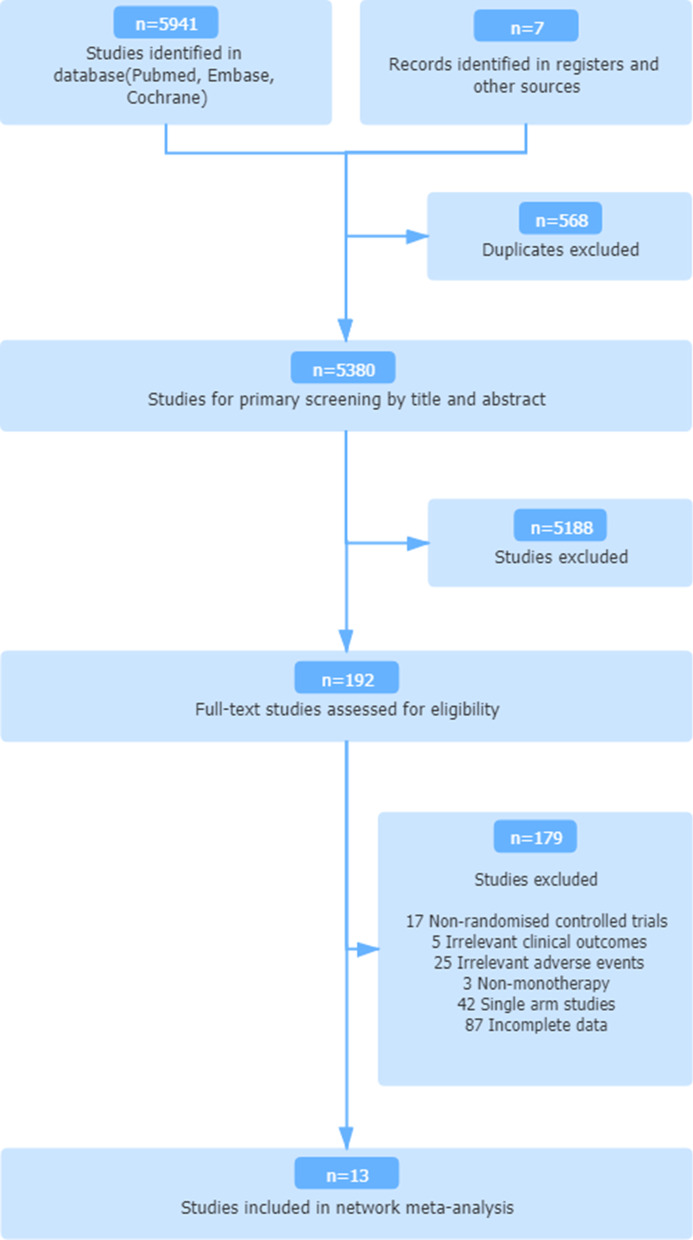
Study selection

**Fig. 2:**
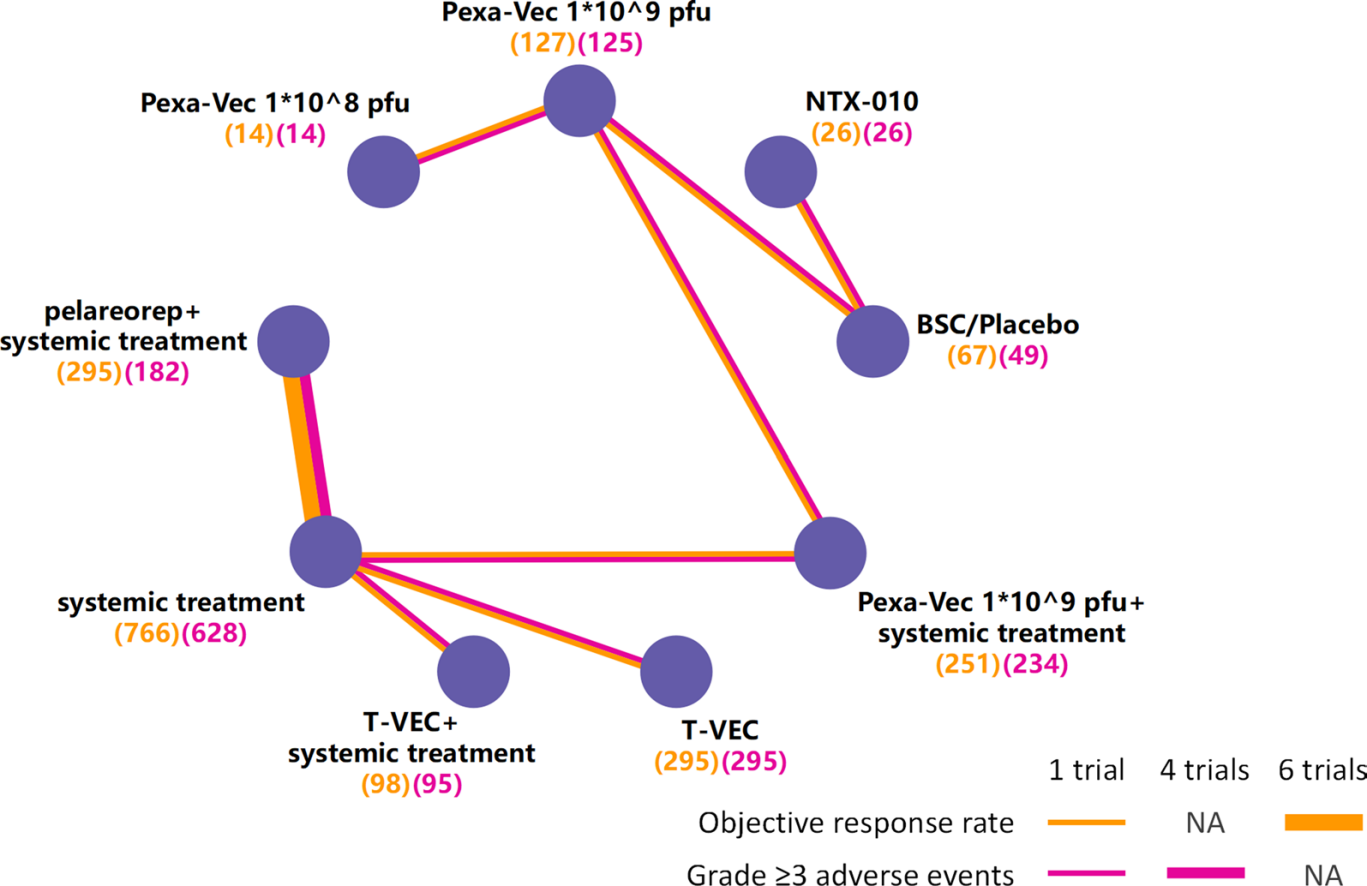
Network plots of comparisons of the ORRs and grade ≥ 3 adverse events for treatments in patients with cancer. Each round node represents one single treatment. The sample size of patients was shown in brackets. Each line represents a type of head-to-head comparison. The width of the lines is proportional to the number of trials comparing the connected treatments (pfu = plaque-forming units)

The titles and abstracts of a total of 5948 records identified from the databases were screened (Fig. 1). Consequently, 13 randomized controlled trials and nine treatments, including four oncolytic viruses (NTX-010, pexastimogene devacirepvec (Pexa-Vec), T-VEC, and pelareorep), were included in the study [13–25]. The 13 studies were shown in Fig. 2. Among the 13 studies with 1939 patients in total, 13 reported ORRs, and 11 reported grade ≥ 3 adverse events. Table 1 summarizes the major characteristics of these trials, including the patient populations and group interventions. Races were not mentioned in most studies either on the website of Clinicaltrials.gov or the published articles. The risk of bias for all trials was assessed and summarized in supplementary Additional file 1: figure S1.

**Table 1:** Baseline characteristics of studies included in the network meta-analysis of patients with cancer (NG = not given, pfu = plaque-forming units, TCID = tissue culture infective dose)

Study (Phase, ID)	Study centres(No)	Sample size (No); median age	Sex (male/female,%)	Study objective	Virus species	Group Intervention (arm 1/arm 2/arm 3/arm 4)
Moehler et al. [13] (II, NCT01387555)	38	86/43;NG	81.4/18.6	Advanced hepatocellular carcinoma	Vaccinia	Pexa-Vec 1 * 10^9^ pfu/best supportive care
Noonan et al. [14] (II, NCT01280058)	6	36/37;64	56.2/43.8	Recurrent or metastatic pancreatic cancer	Reovirus	Pelareorep 3 * 10^10^ TCID 50 plus paclitaxel or carboplatin/paclitaxel or carboplatin
Gedeon et al. [15] (II, NCT01199263)	36	54/54;NG	0/100.0	Recurrent or persistent ovarian epithelial, fallopian tube, or primary peritoneal cancer	Reovirus	Paclitaxel/p pelareorep 3 * 10^10^ TCID 50 plus paclitaxel
Heo et al. [16] (II, NCT00554372)	9	14/16;64.9	76.7/23.3	Unresectable primary hepatocellular carcinoma	Vaccinia	Pexa-Vec 1 * 10^8^ pfu/Pexa-Vec 1 * 10^9^ pfu
Bernstein et al. [17] (II, NCT01656538)	7	36/38;NG	0/100.0	Metastatic breast cancer	Reovirus	Pelareorep 3 * 10^10^ TCID 50 plus paclitaxel/paclitaxel
Bradbury et al. [18] (II, NCT01708993)	12	38/37/39/38;NG	50.7/49.3	Advanced or metastatic non-small-cell lung cancer	Reovirus	Pelareorep 4.5 * 10^10^ TCID 50 plus pemetrexed/pemetrexed/pelareorep 4.5 * 10^10^ TCID 50 plus docetaxel/docetaxel
Chesney et al. [19] (II, NCT01740297)	40	98/100;NG	57.6/42.4	Advanced unresectable melanoma	Herpes simplex	T-VEC 1 * 10^6^ pfu/1 * 10^8^ pfu plus ipilimumab/ipilimumab
Eigl et al. [20] (II, NCT01619813)	11	41/44; 69	NG	Metastatic castration resistant prostate cancer	Reovirus	Pelareorep 3 * 10^10^ TCID 50 plus docetaxel/docetaxel
Jonker et al. [21] (II, NCT01622543)	10	51/52;60	61.2/38.8	Metastatic colorectal cancer	Reovirus	Pelareorep 3 * 10^10^ TCID 50 plus FOLFOX6 or bevacizumab/FOLFOX6 or bevacizumab
Andtbacka et al. [22] (III, NCT00769704)	83	295/141;63.1	57.2/42.8	Unresectable melanoma	Herpes simplex	T-VEC 1 * 10^6^ pfu/1 * 10^8^ pfu/GM-CSF 125 μg/m^2^/day
Schenk et al. [23] (II, NCT01017601)	196	26/24;63	48.0/52.0	Extensive-stage small cell lung cancer	Seneca valley virus	NTX-010 1 * 10^11^ (viral particles/kg)/placebo
PHOCUS [24] (III, NCT02562755)	142	234/225;60.9	84.1/15.9	Advanced hepatocellular carcinoma	Vaccinia	Pexa-Vec 1 * 10^9^ pfu plus sorafenib/sorafenib
Burke et al. [25] (II, NCT01394939)	11	25/17;58.9	40.0/60.0	Metastatic, refractory colorectal carcinoma	Vaccinia	Pexa-Vec 1 * 10^9^ pfu/Pexa-Vec 1 * 10^9^ pfu plus irinotecan

### Network meta-analysis in the consistency model

Figure 2 shows 13 randomized controlled trials evaluating the ORRs and grade ≥ 3 adverse events for nine treatments. The numbers of each adverse event in relation to the incidence are presented in Additional file 1: figure S2.

**Fig. 3:**
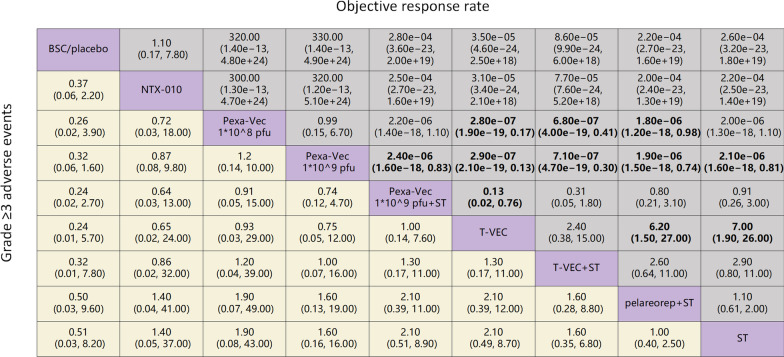
Pooled estimates of the network meta-analysis. Odds ratios (95% confidence intervals) for objective response rates (upper triangle) and grade ≥ 3 adverse events (lower triangle). Data in each cell are the comparison of row-defining treatment versus column-defining treatment. Significant results are shown in bold (BSC = best supportive care, ST = systemic treatment, pfu = plaque-forming units)

In terms of ORR (Fig. 3), T-VEC plus systemic treatment tended to perform better than all other OVs plus systemic treatment (versus pelareorep plus systemic treatment OR 2.60, 95% CI 0.64–11.00, Pexa-Vec 1 * 10^9^ pfu plus systemic treatment versus OR 0.31, 95% CI 0.05–1.80), whereas T-VEC monotherapy also provided a better ORR than other OV monotherapies (Pexa-Vec 1 * 10^9^ pfu versus OR 2.90e−07, 95% CI 2.10e−19–0.13, Pexa-Vec 1 * 10^8^ pfu versus OR 2.80e − 07 95% CI 1.90e−19–0.17, NTX-010 versus OR 3.10e−05 95% CI 3.40e−24–2.10e+18). However, compared with systemic treatments, only three treatments were observed with ORs higher than 1 (T-VEC versus OR 7.00 95% CI 1.90–26.00, T-VEC plus systemic treatment versus OR 2.90 95% CI 0.80–11.00, pelareorep plus systemic treatment versus OR 1.10, 95% CI 0.61–2.00). Furthermore, no significant differences were found between Pexa-Vec 1 * 10^8^ pfu and Pexa-Vec 1 * 10^9^ pfu (OR 0.99, 95% CI 0.15–6.70), Pexa-Vec 1 * 10^9^ pfu plus systemic treatment and systemic treatments (OR 0.91, 95% CI 0.26–3.00), pelareorep plus systemic treatment and systemic treatments (OR 1.10, 95% CI 0.61–2.00), or best supportive care (BSC)/placebo and NTX-010 (OR 1.10, 95% CI 0.17–7.80).

In terms of grade ≥ 3 adverse events (Fig. 3), T-VEC monotherapy (BSC/placebo versus OR 0.24, 95% CI 0.01–5.70) and Pexa-Vec 1 * 10^9^ pfu plus systemic treatment (BSC/placebo versus OR 0.24, 95% CI 0.02–2.70) were demonstrated to cause the most severe adverse events compared to BSC/placebo among the OVs, and these two agents were comparable (OR 1.00, 95% CI 0.14–7.60). From the chart, it can be seen that pelareorep plus systemic treatment was consistent with systemic treatments (OR 1.00, 95% CI 0.40–2.50), whereas other OV therapies were found with ORs higher than 1 relative to systemic treatments. In the individual analysis of safety, five categories of adverse events, including fever, fatigue, diarrhoea, limb oedema, and flu-like symptoms, were recorded as the most common adverse events. The results of network analyses showing odds ratios based on each specific adverse event are presented in Additional file 1: table S4.

### Rank probabilities

**Fig. 4:**
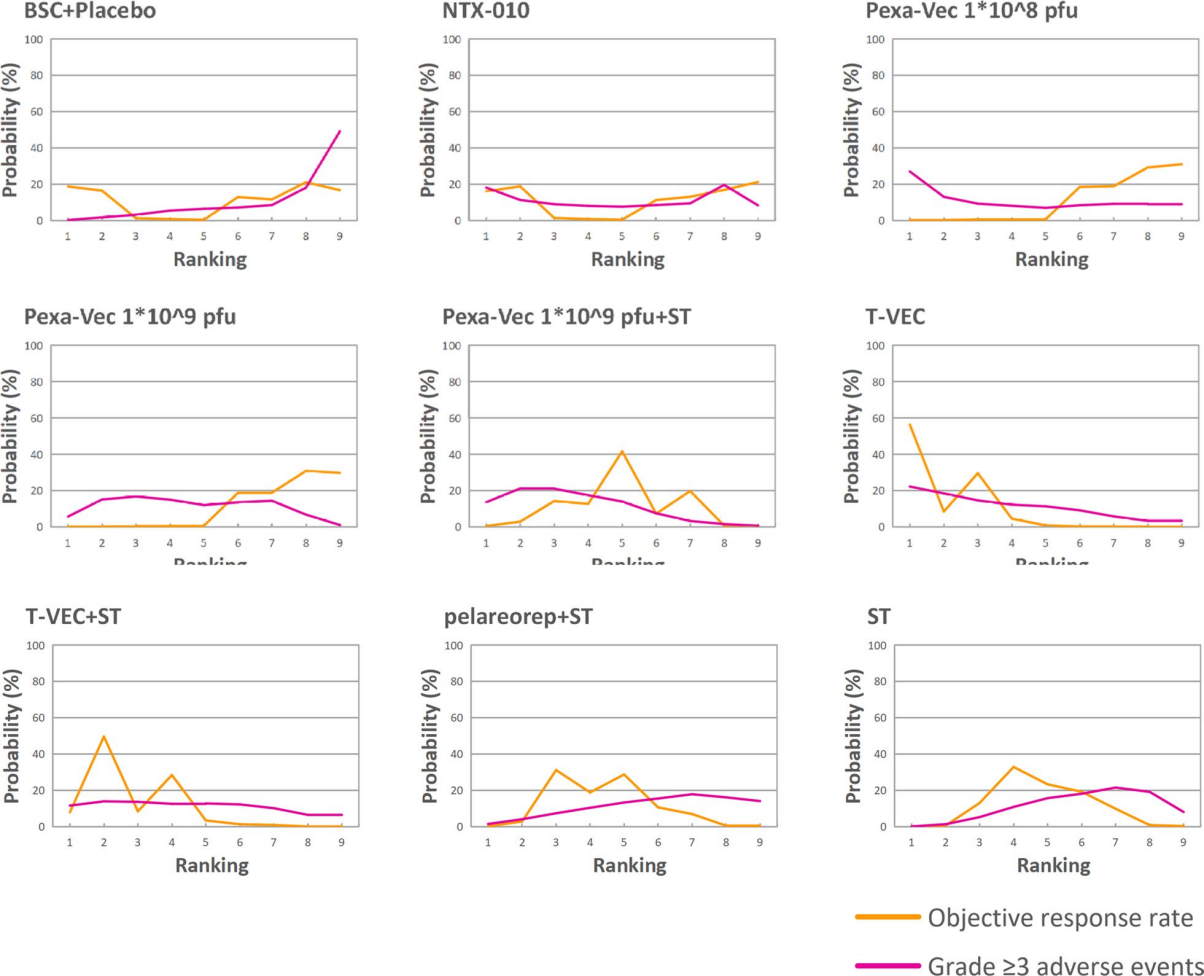
Bayesian ranking probabilities of comparable treatments in terms of efficacy and safety for patients with cancer. Profiles indicate the probability of each comparable treatment being ranked from best to worst in terms of ORRs and grade ≥ 3 adverse events. Ranking sources are described in Additional file 1: table S3 (ST = systemic treatment, pfu = plaque-forming units)

Figure 4 illustrates the Bayesian ranking probabilities of ORRs and grade ≥ 3 adverse events among the nine different treatments. The details of the ranking source are summarized in Additional file 1: table S3. All ranking probabilities were calculated based on the ORs mentioned above. In terms of ORRs, the treatments providing the best ORRs were T-VEC (probability 56%) and T-VEC plus systemic treatment (50%) as monotherapy and combination therapy, respectively. Pexa-Vec 1 * 10^9^ pfu plus systemic treatment (42%), Pexa-Vec 1 * 10^9^ pfu (31%), Pexa-Vec 1 * 10^8^ pfu (31%), and NTX-010 (13%) failed to demonstrate a better ranking than systemic treatments alone. In terms of grade ≥ 3 adverse events, the ranking from worst to best according to probability was as follows: T-VEC (24%), Pexa-Vec 1 * 10^9^ pfu plus systemic treatment (21%), Pexa-Vec 1 * 10^9^ pfu (17%), T-VEC plus systemic treatment (13%), pelareorep plus systemic treatment (13%), systemic treatment (18%), Pexa-Vec 1 * 10^8^ pfu (12%), NTX-010 (20%), and BSC/placebo (49%).

**Fig. 5:**
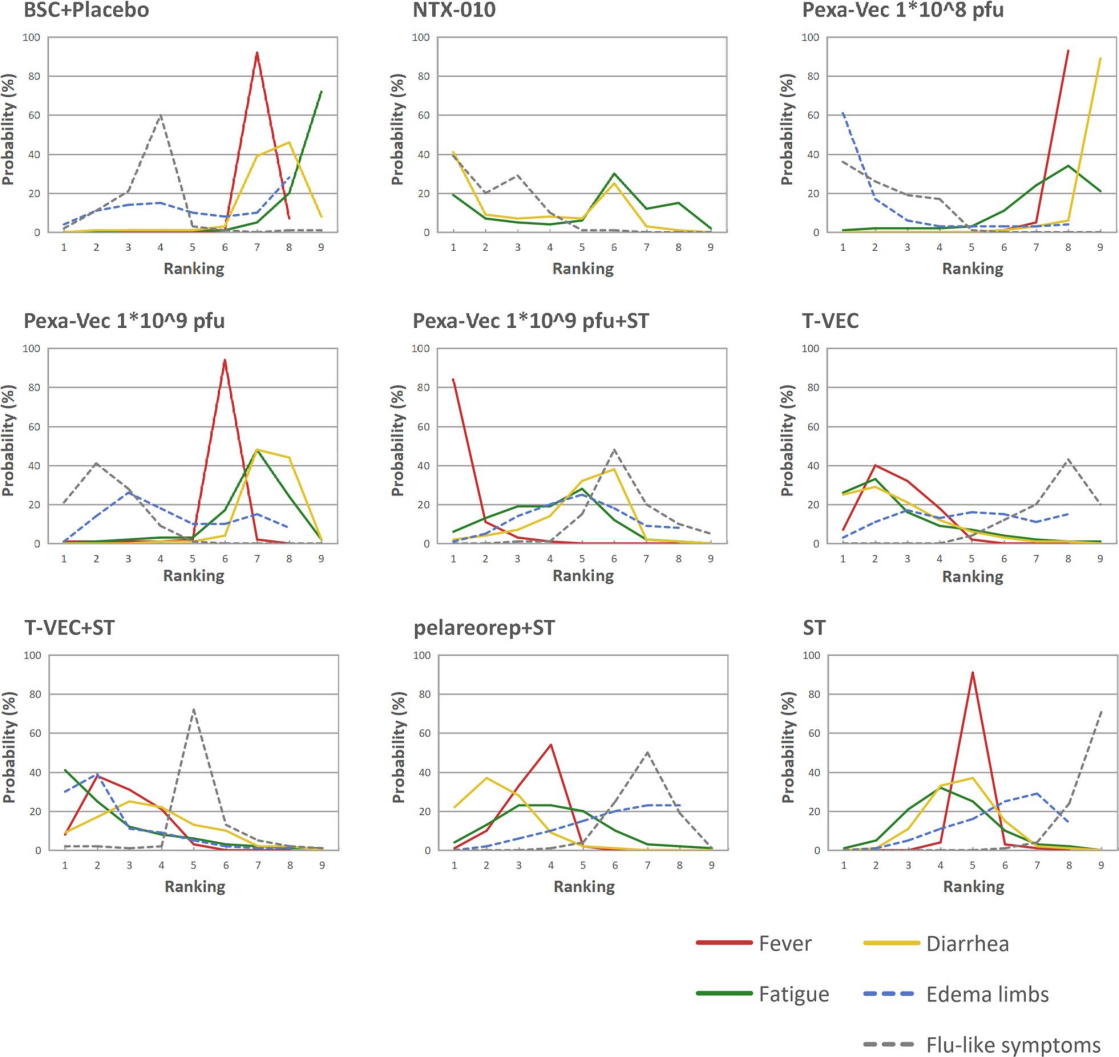
Bayesian ranking probabilities of comparable treatments for each adverse event. Profiles indicate the probability of each comparable treatment being ranked from worst to best on adverse events. Ranking sources are described in Additional file 1: table S5 (ST = systemic treatment, pfu = plaque-forming units)

Figure 5 illustrates the Bayesian ranking probabilities of comparable treatments on individual adverse events. The details of the ranking source are summarized in Additional file 1: table S5. In terms of fever, the rank in the sequence of worst to best was as follows: Pexa-Vec 1 * 10^9^ pfu plus systemic treatment (probability 84%), T-VEC (40%), T-VEC plus systemic treatment (31%), pelareorep plus systemic treatment (54%), systemic treatment (91%), Pexa-Vec 1 * 10^9^ pfu (94%), BSC/placebo (92%), and Pexa-Vec 1 * 10^8^ pfu (93%). T-VEC plus systemic treatment and T-VEC were found to be the worst two agents for fatigue, with probabilities of 41% and 33%, respectively. The treatments inducing the most severe adverse events of diarrhoea, limb oedema, and flu-like symptoms were T-VEC (probability 25%), Pexa-Vec 1 * 10^8^ pfu (61%), and NTX-010 (39%).

### Assessment of heterogeneity and inconsistency

The heterogeneity of ORR and grade ≥ 3 adverse event data was estimated and is presented in Additional file 1: figure S4, where Ι^2^ values greater than 25%, 50%, or 75% indicated low, moderate, or high heterogeneity, respectively [26]. The forest plots illustrated moderate heterogeneity in the ORR (2.4%) and grade ≥ 3 adverse event (50.0%) network comparison of pelareorep plus systemic treatment versus systemic treatment alone. The comparisons of the variance of consistency and inconsistency model are presented in Additional file 1: table S7. The node splitting model was not applicable in this network meta-analysis.

### Sensitivity analysis

With a total of 1787 patients, 12 trials were included in the first sensitivity analysis for the ORR (Additional file 1: figure S3 and Additional file 1: table S6). The Bradbury et al. study (NCT01708993) [18] was excluded due to the potential high risk of selective reporting bias, where the four intervention arms were stratified into two arms without eligible detailed data. As a result, no relevant deviations were observed compared to the original network meta-analysis.

In the second sensitivity analysis for grade ≥ 3 adverse events, a total of 1866 patients and 12 trials were included. The Noonan et al. study (NCT01280058) [14] was excluded due to the 100% rates of grade ≥ 3 adverse events in both arms. Given consideration and assessment, high detection and reporting bias might exist. However, Pexa-Vec 1 * 10^8^ pfu had a probability of 27% to be ranked worst for grade ≥ 3 adverse events instead of ranking seventh from the original analysis.

## Discussion

### Principal findings

In the network meta-analysis of oncolytic virus therapies, 13 trials for patients with advanced or metastatic cancer were evaluated. The general results suggest the following:

1. Among the OV monotherapies, T-VEC was most likely to provide the best ORR for patients with advanced or metastatic cancer but was correlated with the most severe grade ≥ 3 adverse events. Dosing T-VEC alone showed a better ORR than T-VEC plus systemic treatment.
2. Combining OV (Pexa-Vec 1 * 10^9^ pfu or pelareorep) with chemotherapy or target agents was demonstrated to have an ORR that was consistent with that of chemotherapy or target agents alone.
3. Combining OV (T-VEC) with an immune-checkpoint inhibitor (ICI) seemed to provide a better ORR than the combination of an OV (Pexa-Vec 1 * 10^9^ pfu or pelareorep) and chemotherapy/target agents.
4. Compared to systemic treatment alone, the combination of an OV (Pexa-Vec 1 * 10^9^ pfu or pelareorep) and systemic treatment did not significantly increase the rate of grade ≥ 3 adverse events.

An inspiring fact is that a series of studies have demonstrated the efficacy of combining an OV and an ICI. As reported in a previous study [27], oncolytic virotherapy seemed to improve the immune response to anti-programmed death 1 (PD-1) agents by changing the tumour microenvironment. In several cell lines in cancers, increased CD8^+^ T cells, elevated interferon-γ gene expression, and anti-programmed death-ligand 1 (PD-L1) protein expression have been proven. In this phase Ib clinical trial, an ORR of 62% for the combination of T-VEC and pembrolizumab was observed in metastatic melanoma. In addition, a preclinical study demonstrated that combination therapy with localized intratumoural therapy of Newcastle disease virus and systemic cytotoxic T-lymphocyte-associated protein-4 (CTLA-4) blockade caused lymphocytic infiltrates, especially seen in tumour-specific CD4^+^ and CD8^+^ T cells, and anti-tumour effects in distant tumours even without distant viral spread [28]. These studies suggested that OV therapy could enhance the tumour susceptibility to systemic therapy with immunomodulatory antibodies, which might guide treatment choice and optimize future trial designs for investigations of such combination therapies.

T-VEC, an HSV (herpes simplex virus)-1-derived engineered attenuated oncolytic virus, was modified for the deletion of ICP34.5, the HSV-1 gene product mediating neurovirulence and latent infection; thus, T-VEC was not capable of growing within neurons or causing latent infection [29, 30]. Furthermore, copies of the human GM-CSF gene were artificially inserted into the virus, replacing ICP34.5 and providing high levels of expression. The release of GM-CSF can induce the recruitment of dendritic cells and then enhance the immune response to tumour antigens [31]. It was indicated that the combination of an OV and GM-CSF was particularly effective, as lytic cell death correlated with viral replication, sequentially releasing tumour antigens to induce a GM-CSF-enhanced immune response. In a phase II clinical trial [19], the individual lesion-type analysis revealed that responses occurred in both injected and distant tumour burden, and a higher rate of complete reduction in tumour burden (T-VEC + ipilimumab, 23%; ipilimumab, 0%) was observed. A possible explanation for the results might be related to the T-cell-associated immune response. Improved antigen presentation and T-cell priming are characteristics of T-VEC modification, whereas CTLA-4 blockade with ipilimumab promotes T-cell expansion [32]. Therefore, combining these therapies may lead to the enhancement of antitumour immune responses and thereby provide greater antitumour activity than either monotherapy. On the other hand, T-VEC showed the most severe grade ≥ 3 adverse events compared with BSC/placebo. Although the findings in the OPTiM study demonstrated that both T-VEC and GM-CSF were well tolerated, without any treatment-related death events [32], previous studies described flu-like symptoms such as pyrexia, chills, and fatigue as the most common adverse events with T-VEC treatment [33, 34].

One unanticipated finding in our network meta-analysis was that combining Pexa-Vec 1 * 10^9^ pfu or pelareorep with chemotherapy or target agents did not improve the ORR compared with systemic treatments alone, but there was an increased ORR observed when combining T-VEC with ICIs compared with ICIs alone, which indicated that OVs might be more effective when applied with ICIs rather than chemotherapy or target agents. Pexa-Vec, a genetically modified vaccinia virus, is capable of inactivating the viral gene encoding thymidine kinase and expressing human GM-CSF and β-galactosidase. To date, there are no eligible results of studies on Pexa-Vec plus ICIs for cancer. However, the results of the TRAVERSE study [13], a randomized phase IIb trial of Pexa-Vec plus BSC versus BSC care alone in patients with advanced hepatocellular carcinoma refractory to sorafenib, suggested that Pexa-Vec did not improve OS or the ORR as second-line therapy after sorafenib failure. Furthermore, the PHOCUS study [24] showed ORRs of 19.2% and 20.9% in Pexa-Vec plus sorafenib and sorafenib alone, respectively, in which the ORR for Pexa-Vec plus sorafenib was even slightly lower.

### Strengths and limitations

Compared with the reported meta-analyses of OV therapies, this network meta-analysis had several strengths [35–37]. To date, there has been no network meta-analysis to comprehensively describe the efficacy and safety of optional OV therapies; thus, our study established comparisons among all eligible OV monotherapies and combination therapies. As this meta-analysis included and analysed the most recent versions of results and previously unpublished data, potential mistakes caused by various combinations of treatments were prevented. Previous meta-analyses have tended to report the ORR, progression-free survival, overall survival and adverse events by enrolling a series of pairwise comparisons, so these studies failed to make categorical comparisons among agents without eligible clinical trials. In this study, we avoided reviewing survival outcomes because cancers of various systems were included, but the analysis of ORRs was feasible. Furthermore, to enlarge the group scale, these mentioned studies stratified patients receiving a range of OV therapies into one group, showing differences when compared with patients receiving traditional treatment but not providing a detailed analysis of individual OV therapies. We managed to construct a comprehensive network revealing the difference in efficacy and safety in combining OVs with chemotherapy, targeted agents or ICIs, which was also the most important point that clinicians are concerned about.

Several limitations of our study should be stated. First, several agents in our study were stratified to achieve the maximal network and sample size; thus, heterogeneity and bias might exist among the trials. Second, moderate heterogeneity was observed in the ORR analysis, which could be related to the difference in the number of patients enrolled in the studies. Third, the accuracy of the network meta-analysis lies in the reporting quality of enrolled trials. Among the 13 studies, 11 were identified as phase II, and only two of them reached phase III. Comparisons among several OVs (NTX-010, T-VEC, and Pexa-Vec 1 * 10^8^ pfu) were based on solitary pairwise comparisons, which mainly limited the sample size. Therefore, these results might change when further studies are completed. Finally, although the analysis of ORRs is affected less than the analysis of survival outcomes due to the inclusion of cancers in various systems, there could be inevitable heterogeneity.

## Conclusions

In summary, various OV therapies showed different antitumour efficacies and adverse events for patients with advanced or metastatic cancer. Based on this network meta-analysis, T-VEC and T-VEC plus systemic treatment appear to be the best monotherapy and combination therapy, respectively, in terms of ORRs but should be given with caution, paying attention to the possibility of grade ≥ 3 adverse events. On the other hand, combining an OV with chemotherapy or target agents was demonstrated to have efficacy that was comparable to that of chemotherapy or target agents alone. In general, combining OV therapies with ICIs, instead of chemotherapy or target agents, tended to improve the efficacy, but the issue of safety should be considered. These findings could guide treatment choice and optimize future trial designs for investigations of OVs.

## Supplementary Information

**Additional file 1**. **Table S1.** Checklist of the PRISMA extension for network meta-analysis. **Table S2.** Studies search criteria. **Table S3.** The Bayesian ranking results of network meta-analysis. **Figure S1.** Assessment of studies using the Cochrane risk of bias tool. **Figure S2.** Numbers of each adverse events in relation to the incidence (NA = not applicable). **Table S4.** Odds ratios from pooled analysis based on each specific adverse events in any grade where available (NA=not applicable). **Table S5.** The Bayesian results of ranking probability in individual adverse events analysis (NA=not applicable). **Figure S3.** Sensitivity analysis of objective response rate and grade ≥3 adverse events (ST = systemic treatment, pfu = plaque forming units). **Table S6.** The Bayesian ranking results of sensitivity analysis. **Figure S4.** Forest plots showing results of heterogeneity analyzed from pair-wise and network comparison of objective response rates and grade ≥3 adverse events(CI = confidence interval). **Table S7.** The variance results of inconsistency model analysis.

## Data Availability

All data generated or analysed and studies screened during this study are included in the published article. Further details will be provided on the request to correspondence.

## Abbreviations

OV: Oncolytic virus
DAMP: Danger-associated molecular pattern
T-VEC: Talimogene laherparepvec
Pexa-Vec: Pexastimogene devacirepvec
ORR: Objective response rate
GM-CSF: Granulocyte-macrophage colony-stimulating factor
OR: Odds ratio
pfu: Plaque-forming units
BSC: Best supportive care
ST: Systemic treatment
PD-1: Programmed death 1
PD-L1: Programmed death-ligand 1
CTLA-4: Cytotoxic T-lymphocyte-associated protein-4
ICI: Immune-checkpoint inhibitor
